# miR-143-5p as a Prognostic Biomarker in Colorectal Cancer: Inhibition of Tumor Progression Through Targeting MYCBP

**DOI:** 10.5152/tjg.2026.26198

**Published:** 2026-07-21

**Authors:** Chen Mu, Han Zhang

**Affiliations:** 1 Department of Gastroenterology, Fuwai Hospital, Chinese Academy of Medical Sciences and Peking Union Medical College, Beijing, China; 2 Department of Pulmonary and Critical Care Medicine, The Eighth Medical Center of PLA General Hospital, Beijing, China

**Keywords:** Biomarker, colorectal cancer, mechanism, miR-143-5p, MYCBP

## Abstract

**Background/Aims:**

miR-143-5p functions as a tumor-suppressive microRNA across various cancer types. The current study investigated the expression, prognostic value, and role of miR-143-5p in colorectal cancer (CRC).

**Materials and Methods:**

The expression of miR-143-5p was measured by quantitative reverse transcription polymerase chain reaction. Associations between miR-143-5p expression and clinicopathological characteristics of patients with CRC were statistically analyzed. A series of in vitro cell-based assays were performed to investigate the underlying regulatory mechanisms.

**Results:**

miR-143-5p was downregulated in CRC and may serve as a promising independent prognostic biomarker. Reduced miR-143-5p expression was associated with adverse clinicopathological characteristics and decreased 5-year overall survival. MYCBP was identified as a direct target of miR-143-5p. Overexpression of miR-143-5p suppressed the malignant behaviors of CRC cells, which were partially restored by enforced MYCBP expression.

**Conclusion:**

miR-143-5p is downregulated in CRC and may function as a potential independent prognostic biomarker. Moreover, miR-143-5p may play a tumor-suppressive role in CRC by specifically targeting MYCBP.

**Main Points**

- miR-143-5p expression is significantly downregulated in colorectal cancer (CRC) tissues.
- Reduced miR-143-5p expression is associated with adverse clinicopathological characteristics in patients with CRC.
- Low miR-143-5p expression is associated with poor clinical outcomes and adverse prognosis in patients with CRC.
- miR-143-5p suppresses the malignant progression of CRC by directly targeting MYCBP.
- miR-143-5p shows strong potential as a reliable biomarker for prognostic evaluation in patients with CRC.

## Introduction

Globally, the incidence and mortality of colorectal cancer (CRC) continue to increase, making it one of the most common malignancies and a major threat to human health.^1^ In recent years, CRC has shown a trend toward younger onset, which is particularly evident in regions with rapid economic development.^2^ This malignancy severely impairs patients’ physical function and reduces their quality of life.^3^ Despite recent advances in diagnostic approaches and therapeutic regimens, outcomes for patients with CRC remain poor.^4^ The primary cause is the lack of robust biomarkers for early diagnosis, coupled with insufficient understanding of the mechanisms underlying tumor initiation and malignant progression.^5^ Therefore, in-depth characterization of the key molecular mechanisms mediating CRC initiation and progression is critical for identifying novel therapeutic targets and reliable prognostic biomarkers, which may help optimize clinical diagnosis and treatment strategies.

MicroRNAs (miRNAs) play important roles in various biological processes involved in tumorigenesis and cancer progression.^6^ For instance, miR-21 is markedly overexpressed in multiple malignancies, including lung cancer and CRC.^7^^,^^8^ In CRC, distinct miRNA expression profiles are associated with malignant phenotypes and patient prognosis. Empirical evidence has demonstrated that miR-29a-3p functions as a tumor-suppressive miRNA,^9^ whereas reduced miR-9 expression is associated with adverse clinical outcomes in CRC.^10^ Moreover, miR-195-5p may serve as a promising prognostic biomarker for CRC.^11^ Among the miRNAs identified in recent years, miR-143-5p has attracted attention for its key role in various malignancies.^12^^,^^13^ Growing evidence has demonstrated that miR-143-5p expression is decreased in gallbladder cancer.^14^ A recent systematic review also highlighted the key tumor-suppressive role of miR-143-5p in gastrointestinal tumors.^15^ Moreover, emerging studies have shown that miR-143-5p is abnormally expressed in CRC and closely associated with patient prognosis.^16^^,^^17^ However, the expression status, clinical significance, and precise regulatory network of this miRNA in CRC remain to be further investigated. Systematic elucidation of its specific roles and mechanisms in the malignant biological behavior of CRC warrants further investigation in both basic and translational research.

The primary objective of this research is to evaluate miR-143-5p expression in CRC tissues and assess its potential as a prognostic biomarker.

## Materials and Methods

No artificial intelligence or large language model tools were used in the preparation of this manuscript.

### Subjects and Sample Collection

A total of 120 patients with CRC diagnosed at Fuwai Hospital, Chinese Academy of Medical Sciences and Peking Union Medical College between June 2019 and June 2020 were enrolled. All patients had pathologically confirmed CRC, and none had received preoperative radiotherapy, chemotherapy, or other anticancer treatments. During surgery, both cancerous tissue samples and adjacent normal tissue samples were collected. Exclusion criteria included other malignancies, significant hepatic, renal, or other organ dysfunction, autoimmune diseases, and infectious intestinal diseases or ulcerative colitis. This study was approved by the Fuwai Hospital, Chinese Academy of Medical Sciences and Peking Union Medical College’s medical ethics committee (approval no. 2026-3025; approval date:January 23, 2026), and informed consent was duly obtained from patients or their families. Clinical and pathological data were collected from all patients with CRC.

### Quantitative Reverse Transcription Polymerase Chain Reaction Detection

CRC cell lines and patient serum samples were collected for RNA extraction and quantitative analysis. Total RNA was isolated from the harvested cell pellets using TRIzol reagent according to the standard operating protocol provided by the manufacturer. Total RNA from serum samples was isolated using a specialized serum RNA extraction kit to improve the recovery of low-abundance miRNAs and minimize RNA degradation. The purity of RNA was assessed by measuring the optical density (OD)_260_/OD_280_ ratio using a NanoDrop spectrophotometer. cDNA was synthesized using a PrimeScript RT Master Mix (TaKaRa, Dalian, China) and subjected to quantitative reverse transcription polymerase chain reaction (RT-qPCR). Relative gene expression values were calculated and normalized using the 2^⁻ΔΔCt^ comparative threshold cycle method. U6 small nuclear RNA was used as the endogenous internal reference for miR-143-5p normalization, whereas GAPDH was used as the internal reference for MYCBP mRNA expression normalization, to ensure the accuracy and repeatability of the quantitative results. All RT-qPCR experiments were performed in 3 independent biological replicates, each with 3 technical replicates.

### Survival Analysis

All enrolled patients were followed up for 5 years. Follow-up was conducted through regular outpatient visits and standardized telephone interviews, with the primary purpose of recording patient survival status and determining survival endpoints. The final follow-up deadline was June 2025, and all survival data were collected and documented up to this time point.

### Cell Transfection and Cell Model Construction

Human intestinal epithelial cells (HIEC) and CRC cell lines (HCT116, HT29, SW620, and SW480) were cultured in Roswell Park Memorial Institute (RPMI) 1640 medium supplemented with 10% fetal bovine serum (FBS). When cells reached the optimal growth state, 2.0 × 10^5^ cells were seeded into each well of a 6-well plate. Transfection was performed 24 hours after seeding when cells reached approximately 70%-80% confluence. Each well received 1 mL of medium containing 10% FBS, followed by 1 mL of transfection complexes prepared with Lipofectamine 2000 and either miR-143-5p mimics, a miR-143-5p inhibitor, or control miR-143-5p-NC. All transfection experiments were performed in 3 independent biological replicates.

### Cell Counting Kit-8 Assay

Cell proliferation was evaluated using the Cell Counting Kit-8 (CCK-8) assay. Briefly, transfected CRC cells were harvested, resuspended in complete culture medium, and seeded into 96-well plates at a density of 5 × 10^3^ cells per well. Each group included 5 replicate wells, to ensure the reliability of experimental results, and blank wells containing only medium served as background controls. At the indicated time points, 10 µL of CCK-8 reagent was added to each well, avoiding air bubbles during the operation. After incubation for 2 hours, the OD value of each well at 450 nm was measured. The OD values were recorded and used to generate cell proliferation curves, reflecting the growth status and proliferative ability of CRC cells in different groups. Each group contained 3 parallel wells, and the assay was performed in 3 independent biological replicates.

### Transwell Assays

After transfection, CRC cells were harvested and suspended in serum-free RPMI 1640 medium. The cell suspension was added to the upper chambers of Transwell inserts, and the lower chambers were filled with the medium. For invasion assays, the polycarbonate membranes of Transwell inserts were precoated with diluted Matrigel matrix and incubated at 37°C to allow gelation before cell seeding, whereas Matrigel coating was omitted for migration assays. Cells that migrated or invaded to the lower surface of the membrane were fixed with precooled methanol and stained with 0.1% crystal violet solution. After rinsing with PBS to remove excess stain, stained cells were observed and photographed under an inverted microscope for quantitative analysis. Five randomly selected fields were counted per chamber, and all experiments were performed in 3 independent biological replicates.

### Dual-Luciferase Reporter Assay

MYCBP was identified as a putative downstream target gene of miR-143-5p. To validate this targeted interaction, the wild-type and mutated 3′-UTR fragments of MYCBP were amplified by PCR and cloned into the pmirGLO dual-luciferase reporter vector. Cells were then cotransfected with a miR-143-5p mimic or miR-143-5p inhibitor negative control (NC) together with WT or MUT MYCBP 3′-UTR reporter constructs. Luciferase activities were assessed at 48 hours post-transfection, with firefly luciferase signals normalized to Renilla luciferase as the internal control. Each luciferase assay was performed in 3 independent biological replicates, with 3 technical replicates for each biological replicate.

### Statistical Analysis

All statistical analyses were performed using SPSS version 22.0 (IBM SPSS Corp.; Armonk, NY, USA) and GraphPad Prism version 8.0 (GraphPad Software; San Diego, CA, USA). Data are presented as the mean ± SD from 3 independent biological replicates. Comparisons between 2 groups were performed using the Student’s *t*-test. Comparisons among 3 or more groups were performed using one-way analysis of variance (ANOVA) followed by post hoc tests. The association between miR-143-5p expression and clinicopathological characteristics was analyzed using the chi-square test. Survival curves were plotted using the Kaplan–Meier method and compared using the log-rank test. Multivariate Cox regression analyses were performed to identify independent prognostic factors. A *P* value of <.05 was considered statistically significant.

## Results

### Expression of miR-143-5p in Colorectal Cancer

**Figure 1.**
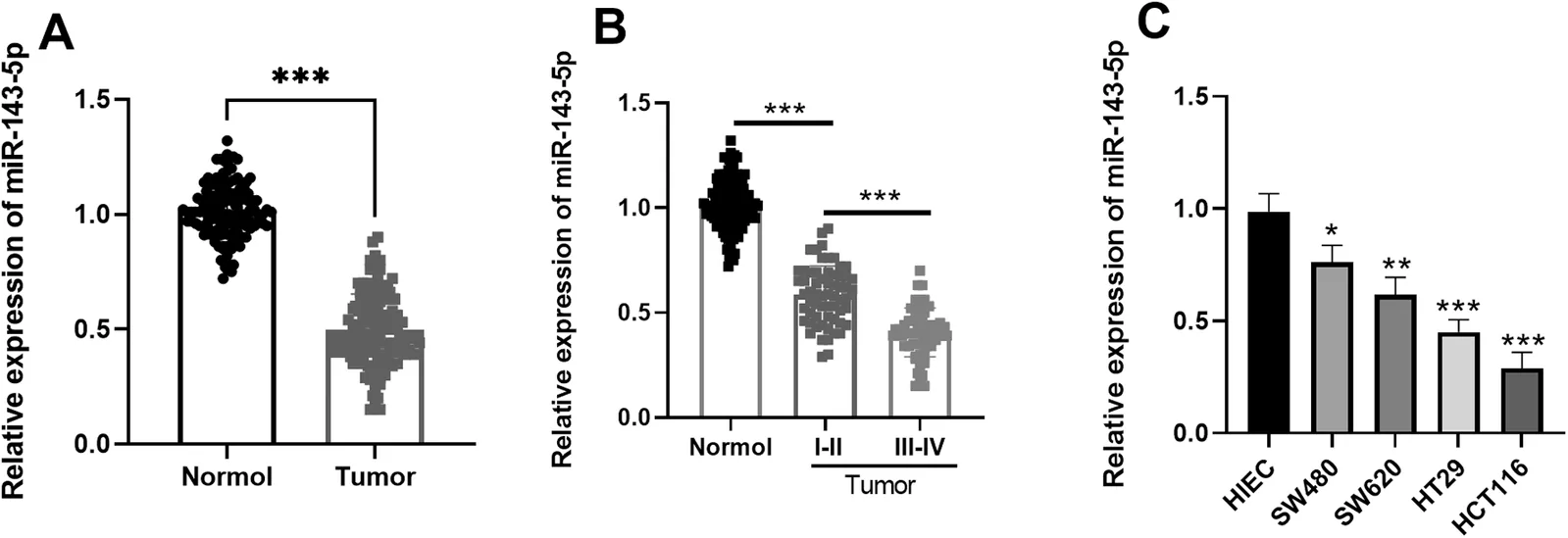
Expression levels of miR-143-5p in colorectal cancer (CRC) tissues and cell lines. (A) Quantitative reverse transcription polymerase chain reaction (RT-qPCR) detection of relative miR-143-5p expression in CRC tissues and adjacent normal tissues. (B) miR-143-5p expression in CRC tissues according to tumor node metastasis stage (I-II vs. III-IV). (C) miR-143-5p expression in normal intestinal epithelial cells (HIEC) and 4 CRC cell lines (HCT116, HT29, SW620, and SW480) determined by RT-qPCR. Data are presented as mean ± SD from 3 independent biological replicates. A paired Student’s *t*-test (A) and unpaired Student’s *t*-test (B, C) were used for statistical analysis. *P<.05, **P<.01, ***P<.001.

Baseline demographic data and clinicopathological characteristics of the study participants are summarized in Supplementary Table 1. The relative expression of miR-143-5p (0.50 ± 0.16) was significantly lower in CRC tissues than in adjacent normal tissues (1.01 ± 0.12, *P*<.001) (Figure 1A). Notably, miR-143-5p expression was considerably lower in patients with stage III-IV disease than in those with stage I-II disease (Figure 1B). At the cellular level, miR-143-5p expression was significantly lower in various CRC cell lines, with the greatest reductions observed in HCT116 and HT29 cells (*P* < .001) (Figure 1C).

### Relationship of miR-143-5p Level with Clinicopathological Parameters

Based on the median expression level of miR-143-5p, 120 patients with CRC were categorized into 2 groups: a low-expression group (n = 63) and a high-expression group (n = 57). Statistical analyses revealed significant correlations between miR-143-5p expression and various clinical parameters, including tumor differentiation (*P* = .039), tumor node metastasis (TNM) staging (*P* = .016), and lymph node metastasis (*P* = .030). In contrast, there was no significant correlation between the level of miR-143-5p and clinical factors including age, sex, tumor location, and invasion depth (*P >* .05) (Table 1).

### Prognostic Role of miR-143-5p in Colorectal Cancer

**Figure 2.**
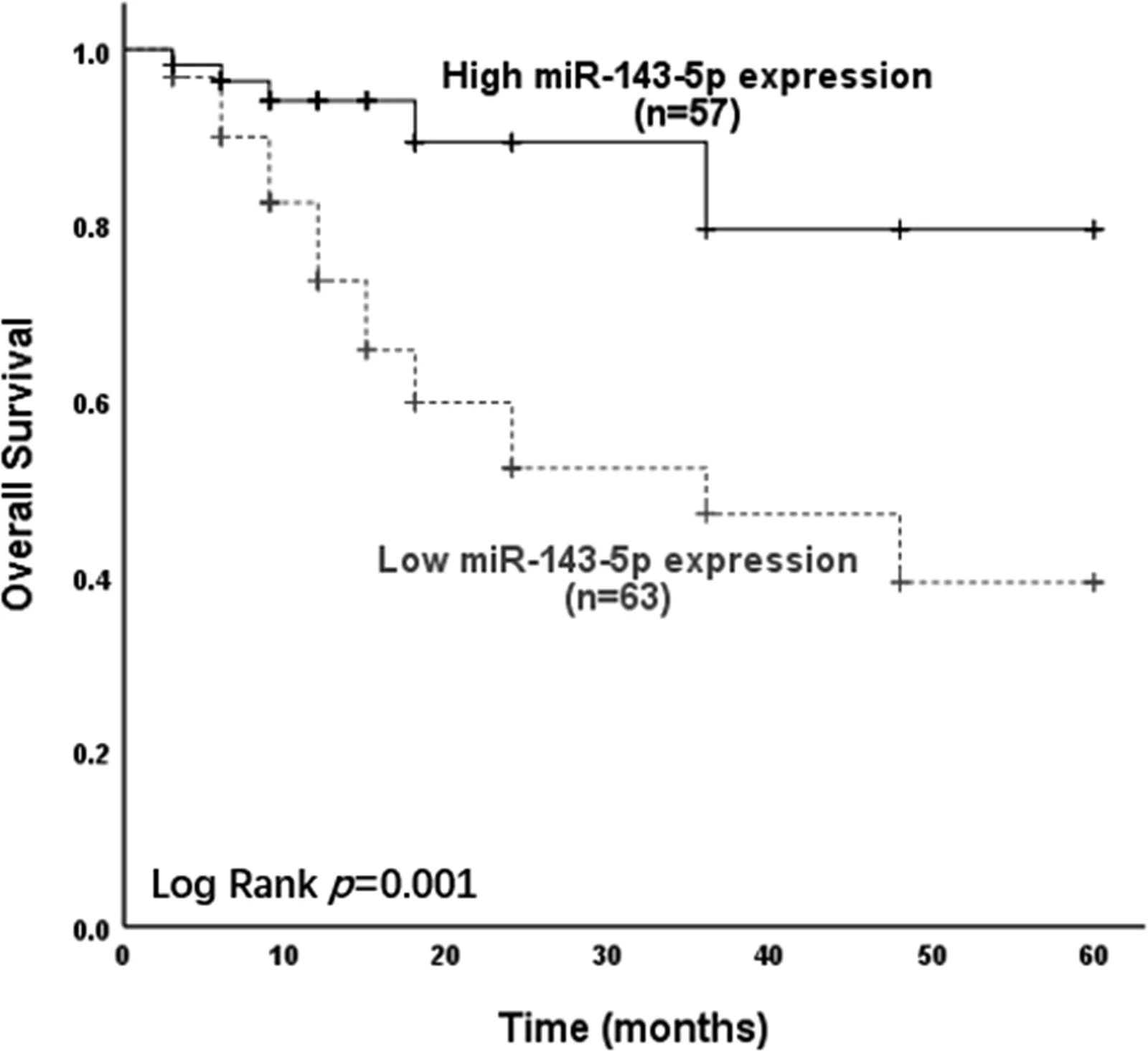
Prognostic significance of miR-143-5p expression in patients with colorectal cancer. Kaplan–Meier survival curve showing the 5-year survival rate difference between miR-143-5p high-expression group and low-expression group. Survival curves were compared by log-rank test. *P* < .05 was considered statistically significant.

Kaplan–Meier analysis demonstrated that patients with low miR-143-5p expression had significantly poorer survival in CRC (*P* = .001) (Figure 2). Furthermore, miR-143-5p expression, TNM stage, and lymph node metastasis were identified as risk factors associated with survival in patients with CRC (Table 2).

### Regulatory Targeting of miR-143-5p to MYCBP

**Figure 3.**
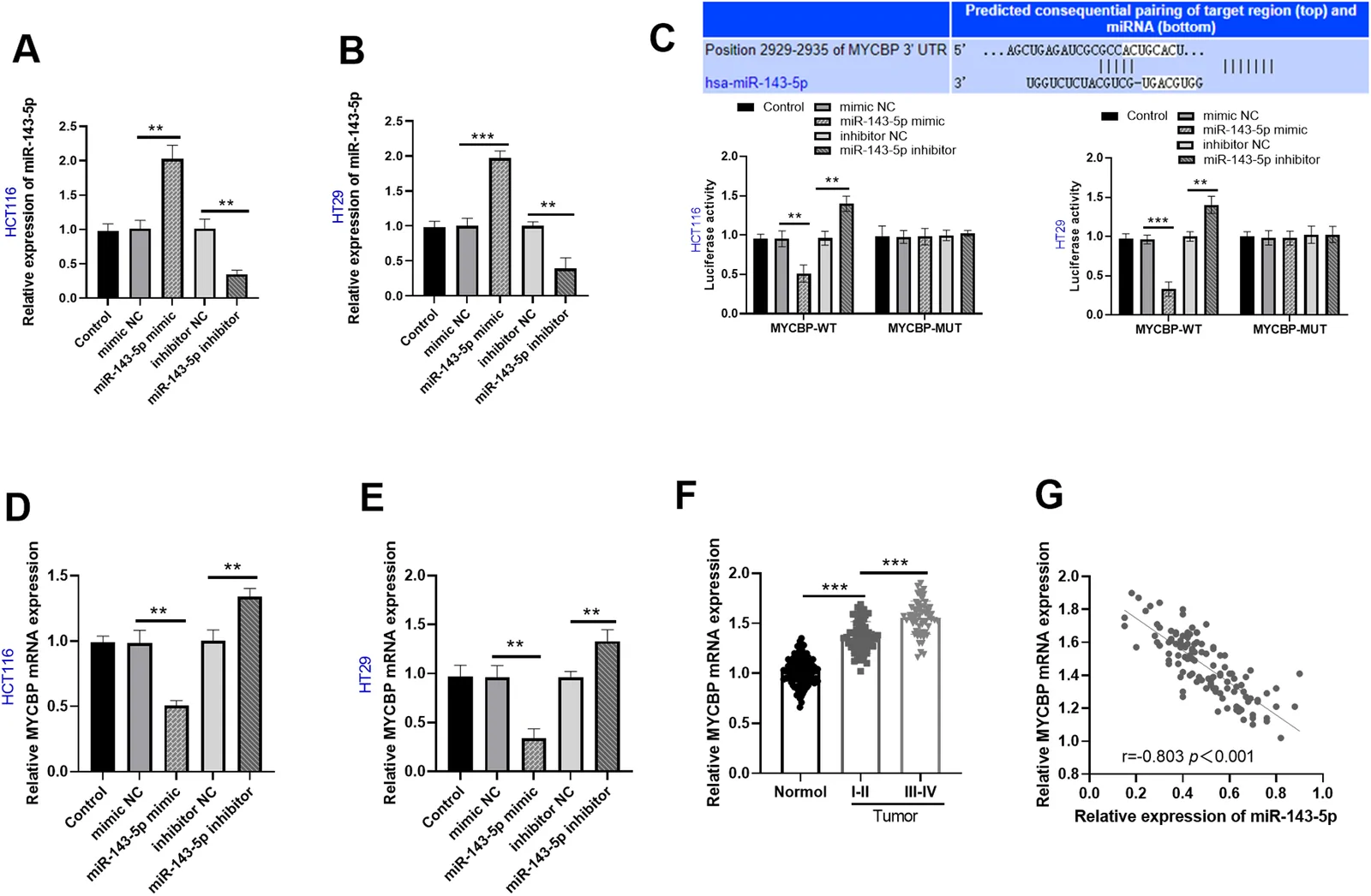
Validation of the targeting relationship between miR-143-5p and MYCBP. (A-B) Quantitative reverse transcription polymerase chain reaction (RT-qPCR) validation of transfection efficiency of miR-143-5p mimic and inhibitor in colorectal cancer (CRC) cells. (C) Dual-luciferase reporter assay showing the binding activity of miR-143-5p to wild-type (WT) 3′-UTR of MYCBP. (D-E) RT-qPCR detection of MYCBP mRNA levels after miR-143-5p overexpression or knockdown. (F) Relationship between MYCBP mRNA expression and tumor node metastasis staging in CRC tissues. (G) Pearson correlation analysis showing a negative correlation between miR-143-5p and MYCBP mRNA expression (*r* = −0.803, *P* < .001). Data are shown as mean porter assay showing the binding activity. Student’s *t*-test was used for statistical comparison. **P < .01, ***P < .001.

Satisfactory transfection efficiency was confirmed, and miR-143-5p overexpression and silencing models were successfully established by cell transfection (Figure 3A-B). The binding sites between miR-143-5p and the 3′-UTR of MYCBP were predicted. Dual-luciferase reporter assays demonstrated that overexpression of miR-143-5p significantly reduced luciferase activity in cells transfected with MYCBP-WT (*P*< .01) (Figure 3C). These findings indicate that miR-143-5p negatively regulates MYCBP expression by binding to the 3′-UTR of MYCBP.

Functional verification experiments demonstrated that enforced overexpression of miR-143-5p decreased MYCBP mRNA expression in CRC cell lines (HCT116 and HT29) (*P*< .05). On the contrary, silencing of miR-143-5p significantly increased MYCBP mRNA expression (*P*< .05) (Figure 3D-E). Analysis of clinical tissue showed that MYCBP mRNA was significantly higher in CRC tissues (*P*< .001). Subgroup analysis showed that MYCBP expression was significantly higher in patients with stage III-IV disease than in those with stage I-II disease (*P*< .001) (Figure 3F). Moreover, miR-143-5p expression was negatively correlated with MYCBP mRNA expression (*r* = −0.803, *P*< .001) (Figure 3G).

### Functional Validation of miR-143-5p/MYCBP Axis in Colorectal Cancer Cells

**Figure 4.**
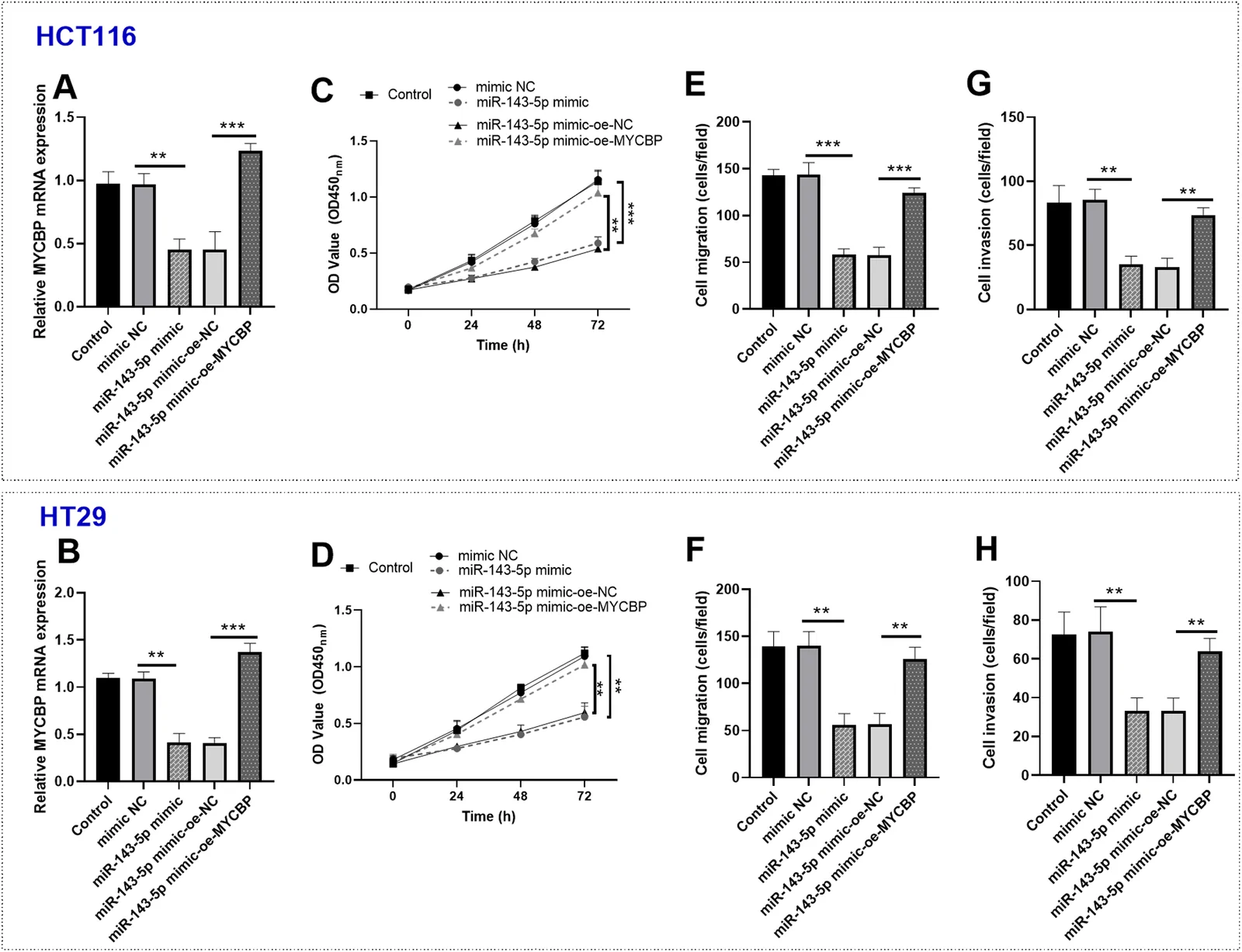
Functional effects of the miR-143-5p/MYCBP axis in colorectal cancer (CRC) cells. (A-B) Quantitative reverse transcription polymerase chain reaction validation of transfection efficiency of MYCBP overexpression plasmid (oe-MYCBP). (C-D) Cell Counting Kit-8 assay showing the proliferation ability of CRC cells after transfection with miR-143-5p mimic alone or combined with MYCBP overexpression plasmid. (E-F) Transwell migration assay and (G, H) Transwell invasion assay evaluating the migratory and invasive capacities of CRC cells in different groups. Data are presented as mean ± SD from 3 independent experiments. One-way ANOVA followed by post hoc test was used for statistical analysis. **P< .01, ***P < .001.

Cotransfection was performed to modulate MYCBP mRNA expression in CRC (HCT116 and HT29) cells (Figure 4A-B). The OD values were remarkably reduced in cells transfected with miR-143-5p mimic (*P* < .05). These findings indicate that miR-143-5p upregulation notably repressed the proliferative ability of CRC cells. Moreover, cotransfection with the miR-143-5p mimic-oe-MYCBP significantly restored cell proliferation capacity compared with cotransfection with the mimic-oe NC group (*P*< .05) (Figure 4C-D). Transwell assays showed that overexpression of miR-143-5p significantly reduced the migratory capacity of CRC cells compared with the negative control group (*P*< .05). Furthermore, restoration of MYCBP expression significantly reversed the inhibitory effects of miR-143-5p on cell migration and invasion (*P*< .05) (Figure 4E-H).

## Discussion

miRNAs have also been reported to participate in CRC progression. For instance, miR-1226-3p is downregulated by the lncRNA MIR210HG, thereby promoting malignant phenotypes of CRC cells.^18^ Consistent with previous evidence, miR-143-5p functions as a tumor suppressor in a wide range of malignancies.^19^^-^^21^ In gastric cancer, miR-143-5p exhibits a more prominent anti-tumor function than its passenger strand miR-143-3p, exerting a stronger tumor-suppressive effect in regulating key malignant phenotypes of cancer cells.^22^ Consistent with previous studies, the findings of the current study confirmed that miR-143-5p was significantly downregulated in CRC tissues, and its reduced expression was associated with adverse clinicopathological features, supporting its role as a tumor suppressor in CRC progression. Furthermore, low miR-143-5p expression was associated with poorer survival, and miR-143-5p was identified as an independent prognostic factor for poor outcomes in patients with CRC. Collectively, the results suggest that miR-143-5p may serve as a prognostic biomarker for CRC.

Emerging evidence indicates that miR-143-5p suppresses breast cancer development and progression by directly targeting HMGA2.^23^ Moreover, restoration of miR-143 expression inhibits the malignant behaviors of CRC cells, further supporting its tumor-suppressive role.^24^ Regarding the underlying molecular mechanisms, miR-143-5p was found to directly target MYCBP and downregulate its expression. Further in vitro functional assays demonstrated that upregulation of miR-143-5p significantly inhibited the malignant biological behaviors of CRC cells. Conversely, rescue experiments demonstrated that restoration of MYCBP expression markedly reversed these inhibitory effects, indicating that MYCBP is a critical functional downstream target mediating the tumor-suppressive role of miR-143-5p. These results suggest the potential role of the miR-143-5p/MYCBP pathway in the development and malignant progression of CRC. Notably, analysis of clinical samples showed that MYCBP was upregulated, whereas miR-143-5p was downregulated in cancer tissues compared with adjacent normal tissues, with a significant negative correlation between them. Collectively, the current data demonstrate that miR-143-5p functions as a critical tumor suppressor in CRC by directly targeting and inhibiting MYCBP, thereby neutralizing its oncogenic activity. The current study deepens the mechanistic understanding of CRC progression and provides supporting experimental evidence for exploring diagnostic biomarkers, prognostic indicators, and targeted therapeutic strategies, with potential scientific value and clinical translational significance.

As a key MYC-binding protein and essential ubiquitin ligase, MYCBP is involved in multiple fundamental cellular processes.^25^^-^^27^ MYCBP functions as an oncogene in CRC, and its upregulation promotes malignant progression.^28^^,^^29^ For instance, miR-26b-5p suppresses breast cancer cell proliferation by directly targeting MYCBP.^30^ However, the specific mechanisms underlying the involvement of MYCBP in CRC remain to be fully elucidated. The findings of the current study demonstrated that miR-143-5p exerts its tumor-suppressive effects by repressing MYCBP expression, which provides new perspectives on the molecular mechanisms underlying CRC progression. In addition, existing evidence has indicated that MYCBP may act as a direct target gene of the β-catenin/LEF-1 signaling pathway.^31^ Earlier studies have shown that MYCBP is a direct target of the β-catenin/LEF-1 pathway through a specific LEF-1 binding site located within the MYCBP promoter. Moreover, overexpression of MYCBP has been reported to enhance c-MYC co-activation in colon cancer cells. Future studies should investigate the downstream signaling cascades of MYCBP, with the aim of elucidating the comprehensive regulatory network of the miR-143-5p/MYCBP axis in CRC.

Collectively, the current findings have potential clinical translational implications for CRC. First, miR-143-5p serves as a promising prognostic biomarker for patients with CRC. Its expression in tumor tissues may aid risk stratification and prognostic assessment, helping clinicians develop individualized therapies and optimize clinical decision-making. Second, the newly identified miR-143-5p/MYCBP regulatory axis provides a potential mechanistic explanation for CRC progression and may serve as a promising molecular target for future research. The design and application of miR-143-5p mimics or inhibitors targeting MYCBP may represent potential approaches for improving the clinical outcome of patients with this malignancy. Furthermore, although the current study focused on tissue miR-143-5p, future studies should investigate its potential as a circulating noninvasive biomarker for diagnosis and prognosis of CRC.

The current study had several limitations: First, validation of the miR-143-5p/MYCBP regulatory axis was limited at the cellular level, lacking complementary in vivo validation using animal models. Second, all clinical samples were obtained from a single center, which may introduce regional and demographic biases. Third, the specific downstream signaling pathways regulated by MYCBP were not investigated. Future studies should use CRC animal models to validate the in vivo effects of miR-143-5p on tumor growth and metastasis and further define the signaling network regulated by MYCBP.

Overall, these findings indicate that the miR-143-5p/MYCBP regulatory axis plays an important tumor-suppressive role in CRC progression. By targeting and suppressing MYCBP expression, miR-143-5p effectively restrains the malignant biological behaviors of CRC cells. Furthermore, the association of miR-143-5p with adverse clinicopathological features and poor survival suggests that the miR-143-5p/MYCBP axis may serve as a prognostic biomarker and potential therapeutic target for CRC, warranting further clinical validation.

## Supplementary Materials

Supplementary Material

## Figures and Tables

**Table 1.** Relationship Between Clinicopathological Characteristics and miR-143-5p Expression Level

Variable	miR-143-5p Expression Level		*P*
	Low (n = 63)	High (n = 57)	
Gender			.667
Male	40	34	
Female	23	23	
Age (years)			.310
<60	26	27	
≥60	37	30	
Tumor location			.126
Colon	31	36	
Rectum	32	21	
Differentiation			.039*
Well/moderate	35	42	
Poor	28	15	
Local invasion			.080
T1/T2	21	28	
T3/T4	42	29	
TNM stage			.016*
I/II	26	36	
III/IV	37	21	
Lymph node metastasis			.030*
Negative	39	24	
Positive	24	33	

CRC, colorectal cancer; TNM, tumor node metastasis.

*P <.05.

**Table 2.** Multivariate Cox Analysis of Survival Factors for Patients with CRC

Variable	HR	95% CI for HR		*P*
		Lower	Upper	
miR-143-5p	0.285	0.098	0.829	.021*
Gender	0.737	0.318	1.710	.477
Age	1.534	0.672	3.498	.309
Tumor location	0.551	0.246	1.234	.147
Differentiation	2.086	0.944	4.608	.069
Local invasion	2.322	0.824	6.547	.111
TNM stage	2.624	1.078	6.391	.034*
Lymph node metastasis	3.056	1.200	7.782	.019*

CRC, colorectal cancer; HR, hazard ratio; TNM, tumor node metastasis.

*P<.05.

## Data Availability

The data that support the findings of this study are available on request from the corresponding author.
