# Supplementary material for: miR-143-5p as a Prognostic Biomarker in Colorectal Cancer: Inhibition of Tumor Progression Through Targeting MYCBP
Source: Turk J Gastroenterol. 2026 Jul 21;37(9):949–57. doi: 10.5152/tjg.2026.26198 (PMC13554479; doi:10.5152/tjg.2026.26198)
Supplement: Supplementary file 1 [file supplementary_material.pdf]

Supplementary Table 1. Baseline clinicopathological characteristics of CRC patients

Characteristic	Category	n
Age (years)	<60	53
	≥60	67
Gender	Male	74
	Female	46
Tumor location	Colon	67
	Rectum	53
Tumor differentiation	Well/moderate	77
	Poor	43
TNM stage	I–II	62
	III–IV	58
Lymph node metastasis	Negative	63
	Positive	57
Local invasion	T1–T2	49
	T3–T4	71

CRC, colorectal cancer; TNM, Tumor Node Metastasis.
